# Numerical and Experimental Investigation of the Conjugate Heat Transfer for a High-Pressure Pneumatic Control Valve Assembly

**DOI:** 10.3390/e24040451

**Published:** 2022-03-24

**Authors:** Mboulé Ngwa, Longlong Gao, Baoren Li

**Affiliations:** FESTO Pneumatic Technology Centre, School of Mechanical Science and Engineering, Huazhong University of Science and Technology, Wuhan 430074, China; ngwa_m@yahoo.fr (M.N.); lbr@hust.edu.cn (B.L.)

**Keywords:** conjugate heat transfer, high-pressure pneumatic control valve, URANS CFD methods, standard *k-ε* turbulence model, polyhedral mesh, local entropy production rate

## Abstract

This paper uses heat transfer experiments and computational fluid dynamics (CFD) simulations to investigate the conjugate heat transfer (CHT) in a high-pressure pneumatic control valve assembly. A heat transfer test rig was constructed, and time–temperature histories of five test points placed on the valve assembly’s outer surface were recorded for study validation. The Unsteady Reynolds-Averaged Navier–Stokes (URANS) CFD methods with the standard *k-ε* turbulence closure equations were adopted in the numerical computations. Polyhedral grids were used; time step and mesh convergence studies were conducted. Simulated and measured temperatures profile comparisons revealed a good agreement. The CHT results obtained from CFD showed huge velocity fields downstream of the valve throat and the vent hole. The airflow through the valve was icy, mainly in the supersonic flow areas. Low temperatures below 273.15 K were recorded on the internal and external walls of the valve assembly. The consistency of the measured data with the numerical results demonstrates the effectiveness of polyhedral grids in exploring the CHT using CFD methods. The local entropy production rate analysis revealed that irreversibility is mainly due to viscous dissipation. The current CHT investigation provides a potential basis for thermostress analysis and optimization.

## 1. Introduction

Pneumatic control valves are essential components of high-pressure pneumatic systems. They are widely applied in defense applications, aerospace, compressed gases and equipment, submarines, etc. They are used to readjust the steam flow rate, airflow, and other compressed gases by opening or closing orifices. The regulation of fluid flow through the valve orifice results in the alteration of several parameters such as the pressure and temperature inside its chamber. When it becomes significant, the pressure drop leads to considerable heat exchange between the transmitted fluid, the walls of the valve assembly, and the surrounding atmosphere. Due to the heat interaction, the uneven temperature distribution generates thermal stresses that significantly reduce the structure’s performance. Understanding these different processes involving temperature changes is vital for high-pressure pneumatic components and systems.

A literature survey shows that various efforts have been made to study the performance of the different types of control valves using computational fluid dynamics (CFD) methods. Amirante et al. [1,2,3,4] investigated the flow properties and the dynamic characteristics of forces acting on the spool of the hydraulic control valve through the CFD approach using the RNG *k-ε* turbulence model and found good agreement with experimental data. They draw our attention to the need to focus on a full three-dimensional (3D) model for studies involving complex flow in control valves. Lafond et al. [5] analyzed 3D complex flow-fields within hot pressure gas based on CFD techniques and compared the simulated results with results of cold flow tests. A CFD study applying the moving grid method has been performed by Srikanth et al. [6] to examine the compressible airflow characteristics in the puffer chamber. In works [7,8], the CFD approach was used to analyze the dynamic flow properties in the servo valves. Lisowski et al. [9] completed a 3D CFD simulation survey of the flow forces acting on the directional control valve adopting the *k-ε* turbulence model closure and carried out experiments to further optimize the valve shape. Increasingly, researchers have become interested in CFD techniques and visualizations to improve the insight on flow in control valves [10,11]. García-Todolí et al. [12] investigated the characteristics of air valves employing the CFD approach. They found good agreement with experimental data, revealing that CFD methods are a valuable option for studying the behavior of air valves. Shiyang et al. [13] studied the dynamic characteristics of a solenoid valve using transient CFD numerical calculations and experiments. Some authors [14,15] used the Reynolds-Averaged Navier–Stokes (RANS) simulation method with the shear stress transport (SST) turbulent closure to analyze flow properties in turbine control valves. In general, these works focused more on the flow properties in valves; they do not address the issues of the combined heat transfer characteristics in fluids and high-pressure pneumatic control valves.

In recent years, studies on the heat transfer efficiencies of high-pressure pneumatic components have gradually become available. Marcin [16,17] proposed a new CFD method based on polyhedral grids for CHT and cyclone in a fluidized bed unit. They demonstrated the relevance of polyhedral meshing in dealing with CHT analysis. However, no experimental studies have been conducted to validate the results of the numerical simulations. The author in [18] adopted the polyhedral mesh to numerically investigate the thermal–hydraulic properties of a twisted oval tube with three cross-sectional wire coils in the turbulent regime. Gao et al. [19] studied the transient heat transfer coefficient performance between the high-pressure air within the chamber and the outside atmosphere based on the combined thermodynamics approach, CFD techniques, and fundamental heat transfer theories. According to their work, the flow was hypersonic downstream of the valve. Lee et al. [20] performed a transient analysis of the conjugate heat transfer in a 2D hot gas valve using the CFD technique along with the one-equation Spalart–Allmaras model selected from the three turbulence models adopted during simulations. Their results indicated a weak temperature difference in the structural domain with significant thermal gradients observed in the flow field downstream of the valve opening. Conducting a combined heat transfer analysis based on the CFD numerical model and experiments is appropriate for studying the gas turbine vane [21]. In most previous works, the investigation focused on the characteristics of the flow rate field and the heat transfer performance within the valve based on the two-dimensional (2D) model. Moreover, research data on the impact of the heat transfer characteristics on the surfaces of the high-pressure pneumatic control valves are still limited, showing the existing gaps in the study of the CHT for high-pressure pneumatic control valves adopting the 3D shape.

The compressed airflow through the valve is associated with crucial effects such as gas expansion, significant temperature drops, heat transfer properties, and fluid friction. These factors are the primary sources of irreversibilities in the heat transfer medium [22]. Entropy production due to irreversible processes using the theory of the second law of thermodynamics is of great concern in the heat transfer process of engineering systems [23]. Considerable studies have been performed to examine the effects of entropy generation in thermal equipment [24,25]. Entropy analysis for heat transfer by forced convection and radiation in a flow-through tube has been performed by Gbadebo et al. [26]. Their results showed that wall temperature and volumetric entropy production increased with the tube length; this rise in entropy creation may indicate an increase in convective heat transfer. In their study, however, fluid friction was ignored. Likewise, Hyder et al. [27] established theoretical methods to investigate the temperature distribution and scrutinize the entropy generation in a flow system, assuming no viscous dissipation in the energy equation. Kock et al. [28] performed a *k-ε* model CFD analysis to study irreversibility sources in turbulent shear flow based on asymptotic considerations and wall functions. They found that the maximum entropy production occurred near the wall and concluded that wall functions are mandatory when high-Reynolds-number turbulent models are used. A literature review of fluids flowing in ducts and channels has documented that entropy production was driven by friction and heat transfer [29,30]. The same observation was made by the author of [31] in his scientific note addressing the total irreversibility in a system. Yaping et al. [32] recently developed a RANS-based CHT method to enhance the prediction of the CHT characteristics and scrutinize the entropy generation analysis by using various turbulent models of the flow properties in the 3D internally cooled gas turbine. The main results of this study indicated that the local entropy creation was mainly confined in the vicinity of the wall and wake regions, and irreversibilities were primarily caused by heat transfer in internal cooling and by friction in the flow passage. Scholars have shown the relevance of entropy creation analysis in engineering sciences. Therefore, studying the sources of irreversibilities in the high-pressure pneumatic control valve during the CHT process is crucial as it will provide designers with valuable details for design improvements.

This study conducts a CHT analysis for a high-pressure pneumatic control valve assembly based on experiments and CFD simulations. A heat transfer experiment was designed at the same scale as the calculation domain, and temperature–time changes were recorded at chosen checkpoints for validation against the simulated results. A 3D model, including the valve assembly and the compressed airfield, was considered for the numerical simulations. Polyhedral cells were selected for mesh generation. Studies of the convergence of the grid and the size of the time steps were conducted. The Unsteady Reynolds-Averaged Navier–Stokes (URANS) CFD method was applied with the standard *k-ε* two-equation turbulence model to scrutinize the flow and heat transfer properties. An investigation of the local entropy generation rate was conducted to provide an insight into the heat dissipation in the flow system. The numerical results are compared with experimental data, the CHT characteristics analyzed, and the conclusion is drawn.

## 2. Structure of the Pneumatic High-Pressure Valve Body and Working Principle

**Figure 1:**
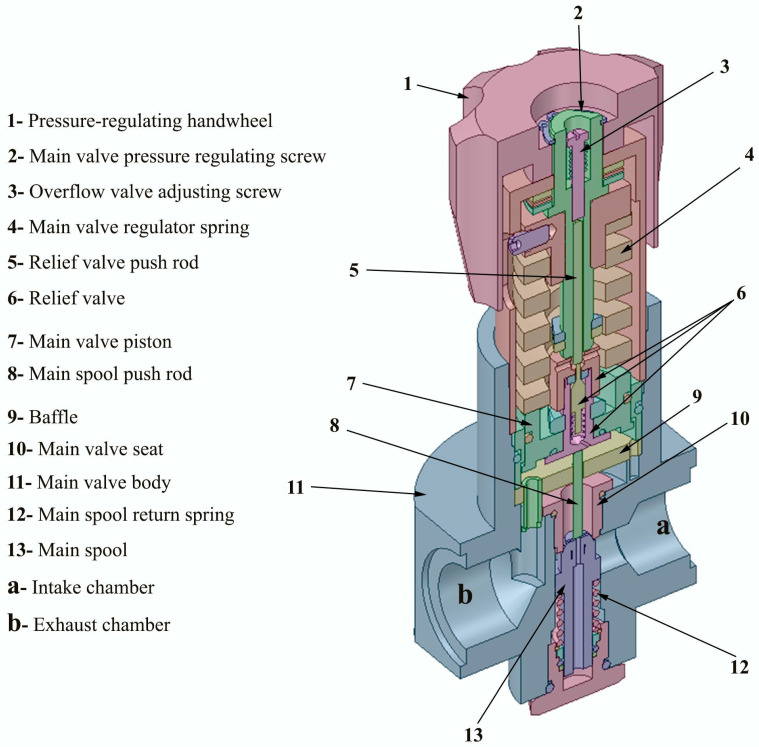
Valve body structure.

Figure 1 shows the structure of the pneumatic high-pressure valve body and its various components. By turning the pressure-regulating handwheel clockwise, the pressure-regulating nut moves downwards to compress the spring, pushing the main valve piston down, which moves the return spring back through the pushrod to open the main valve port. Therefore, compressed air enters inlet chamber **a**, throttles and depressurizes through the valve orifice, and exits through outlet chamber **b**. Once the gas pressure of the exhaust chamber **b** acting on the piston is balanced by the spring force applied by the regulating spring on the valve piston, the opening of the main valve orifice remains unchanged and the outlet pressure of the exhaust chamber **b** is stable.

As the incoming pressure fluctuates or increases instantaneously, the pressure in the exhaust chamber **b** also rises, and the air pressure acting under the main valve piston increases, destroying the initial balance. The piston moves upwards to compress the spring. The return spring also pushes the main spool up, narrowing the opening of the main valve orifice, increasing the throttle resistance, and reducing the pressure of the exhaust chamber **b** until the valve piston reaches the new equilibrium position. Otherwise, the reverse is also true.

If the handwheel is rotated counterclockwise, the spring is relaxed, the air pressure under the main valve piston is greater than the spring force, and the valve piston moves upwards with the overflow valve. The opening of the valve port is reduced until it is entirely closed by the action of the spool return spring. At this time, the top of the overflow valve spool hits the relief valve push rod and moves downward relative to the relief valve, which is no longer in contact with the sealing ring. The valve orifice is opened to pass gas into the exhaust chamber **b** through the main spool to reduce the outlet pressure or return to the zero-installation state. The opening sensitivity of the valve can be adjusted by setting the overflow valve-adjusting screw.

## 3. Experimental Setup and Conditions

**Figure 2:**
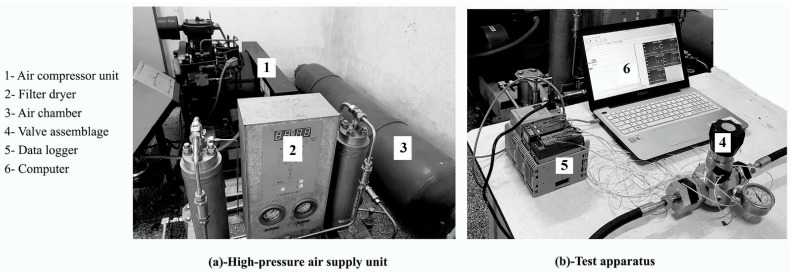
Photograph of the experimental devices.

**Figure 3:**
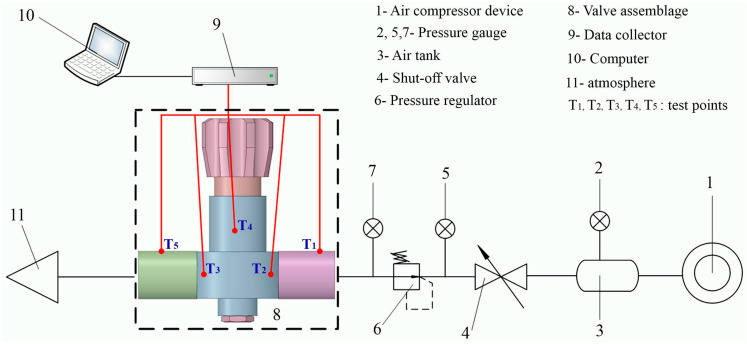
Experimental layout of the conjugate heat transfer.

Figure 2a,b illustrate the photographic representation of the experimental equipment used during the test. The scheme of the experimental setup shown in Figure 3 was designed and implemented to tackle the conjugate heat transfer process. It mainly consists of an air compressor, an air tank, pressure gauges, a pressure regulator, a shut-off valve, a valve assembly and pipes, thermal-resistance sensors (PT100, Shanghai, China), a data collector (Data acquisition module GM10, Yokogawa, Tokyo, Japan), and a computer (Asus R556L). The operating medium is air, which is compressed and stored in the tank at a maximum of 20 MPa. The pressurized air passes through the valve orifice and is released into the atmosphere. Fives thermal-resistance sensors T_1_, T_2_, T_3_, T_4_, and T_5_ of type PT100 with a precision of ±0.1 Kelvin (K) are used to record the transient temperature profiles on the outer surfaces of the pneumatic high-pressure valve assemblage, at the sampling intervals of 1 s. The operating range of the temperature sensor is 323.2 ± 0.1 K to 473.2 ± 0.1 K. Two thermal-resistance sensors, T_1_ and T_5_, are fixed at the upper external surface of the inlet and outlet connector, respectively. The remaining are placed on the outer surface of the valve body. T_2_ is located at the lower cylindrical part of the valve surface and near the valve intake chamber; T_3_ is fixed close to the valve exhaust chamber; T_4_ is set at the upper cylindrical outer surface of the valve body.

The experimental procedure is described as follows:

i. Compress air up to 20 MPa within about 45 min (min);
ii. Link the inlet and outlet connectors to the intake and exhaust valve bodies;
iii. Connect the pressure regulator and the inlet and outlet connector with appropriate pipes;
iv. Secure the valve body with a vice;
v. Attach the five thermal resistance sensors to the selected test points and connect them to the data logger and computer;
vi. Start recording data;
vii. Record the room temperature using the thermal-resistance sensor;
viii. Open the shut-off valve and adjust the input pressure up to 5 MPa;
ix. Set the valve pressure gauge to 0.3 MPa;
x. When the temperature profile reaches equilibrium, stop recording data, close the pressure regulator, then close the shut-off valve;
xi. Repeat the procedure three times, starting from f.

The experiment was conducted in the test room. The initial temperature in the room was about 286.1 ± 0.1 K. The temperature data acquisition starts by recording the room temperature before the beginning of the experiment. The valve opening is set at 20%. Once the control valve is open, the compressed air is regulated to 5 MPa. A pressure gauge placed on the valve body near the outlet chamber allows setting the exit pressure at 0.3 MPa. Then, the temperatures are recorded every second in the computer via the data logger. The test stops once thermal equilibrium is reached, which is after approximately 34 min, and data are saved.

## 4. Numerical Methods

In the present conjugate heat transfer (CHT) investigation, a half geometry was considered to reduce the simulation time due to the complex three-dimensional (3D) computational model and the symmetrical nature of the internal airflow along the *z*-axis. The Unsteady Reynolds-Averaged Navier–Stokes (URANS) computational fluid dynamics (CFD) numerical methods were used to predict the flow field performances and to capture the temperature field characteristics of the high-pressure pneumatic control valve assemblage.

### 4.1. Computational Domain

**Figure 4:**
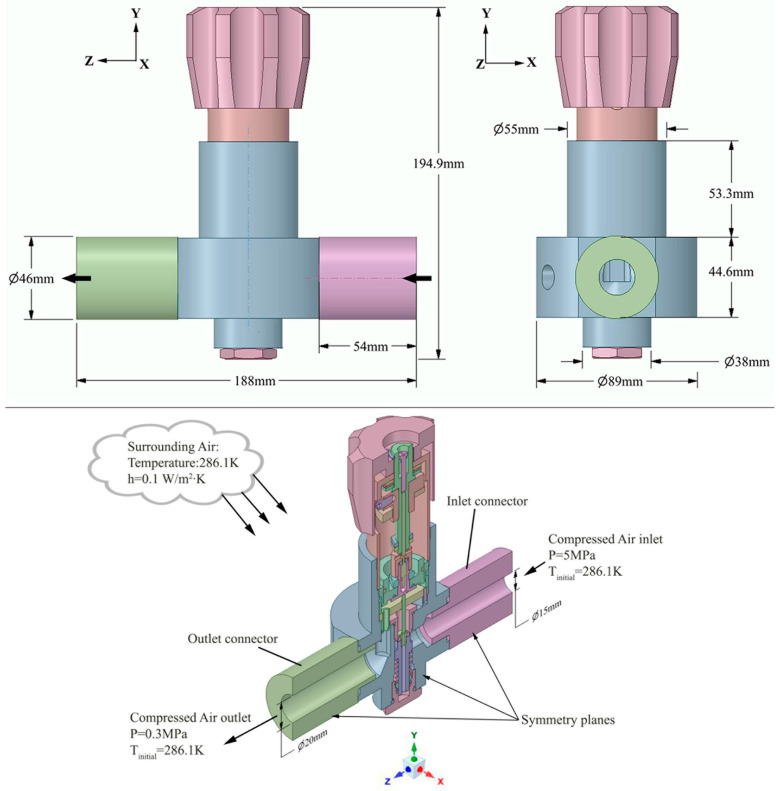
Half 3D computational domain.

Half of the three-dimensional domain shown in Figure 4 was used to enhance the computational efficiency, with a valve opening of 20%. It consists of the main valve body, the inlet and outlet connectors, and the main valve seat. The inlet port has an inner diameter of 15 mm and is fitted to the valve intake chamber. The exhaust chamber of the main valve is secured to the output port, which has an internal diameter of 20 mm. The pressurized air passing through the valve assembly constitutes the inner fluid medium. The computational geometry has been further simplified by removing few components above the piston sleeve, the pressure-regulating handwheel, and the main valve’s top cover.

### 4.2. Mathematical Model

The heat transfer process experienced in the high-pressure pneumatic control valve assemblage comprises two significant phenomena: convection and conduction. At the control valve assembly, the heat exchange involved the convection from the bulk of the compressible airflow to the inner surface of the structural domain, the conduction process through the walls, and the convection from the bulk of the ambient air to the external surface of the valve assembly. The present study solved the CHT problem in the fluid and substantial domains within the same solver and through the fluid–structure interface: Ansys CFD Enterprise software Fluent 19.0. The medium was air. Air flowing inside the structure was considered an ideal gas, treated as a continuous medium, and assumed to behave as a Newtonian fluid.

Moreover, it was regarded as a 3D viscous compressible flow. The change in latent heat and radiative heat transfer were neglected in this investigation. Therefore, the system of equations for the conservation of mass, momentum, and energy was solved in the fixed frame under unsteady state conditions by finite volume method and written as follows [21]:

Continuity Equation:

(1) $$\frac{\partial \left(\rho T\right)}{\partial t}+\frac{\partial \left(\rho U_{i}\right)}{\partial x_{i}}=0,$$

where ρ represents the density of the ideal gas, $U\left(U_{x},U_{y},U_{z}\right)$ is the 3D fluid velocity in the orthogonal Cartesian coordinates, and *t* is the time coordinate.

Momentum Equation:

(2) $$\frac{\partial \left(\rho U_{i}\right)}{\partial t}+\frac{\left(\rho U_{i}U_{j}\right)}{\partial x_{j}}=-\frac{\partial P}{\partial x_{i}}+\left(\mu +\mu_{t}\right)\left[\frac{\partial }{\partial x_{j}}\left(\frac{\partial U_{i}}{\partial x_{j}}\right)+\frac{1}{3}\frac{\partial }{\partial x_{i}}\left(\frac{\partial U_{j}}{\partial x_{j}}\right)\right],$$

where *P* is the medium pressure, μ is the fluid dynamic viscosity, and $\mu_{t}$ is the turbulent viscosity.

Total energy Equation:

(3) $$\frac{\partial \left(\rho T\right)}{\partial t}+\frac{\partial \left(\rho U_{i}T\right)}{\partial x_{i}}=\frac{\partial }{\partial x_{i}}\left(\frac{\lambda_{\mathrm{cg}}}{c_{p}}\frac{\partial \left(T\right)}{\partial x_{i}}\right)+\frac{S_{T}}{c_{p}},$$

where the subscripts i and j are defined as i, j = 1, 2, 3. The cartesian coordinates $x_{1},x_{2},x_{3}$ and the components of the velocity vector $U_{1},U_{2},U_{3}$ are referred to as x,y,z and $U_{x},U_{y},U_{z}$, respectively, with $\lambda_{\mathrm{cg}}$ as the thermal conductivity of the compressed gas, $c_{p}$ as the specific heat at constant pressure, and $S_{T}$ as the energy viscous dissipation term.

It was assumed that air flowing inside the pneumatic control valve assembly followed the perfect gas law. Therefore, the density equation of state was derived and written as described below:

(4) $$\rho =\frac{M\left(P+P_{ref}\right)}{R_{a}T}$$

where $R_{a}$ represents the specific air constant equal to 287.1 J/kg·K.

The heat transfer in the solid domain was achieved by conduction. The simplification of Equation (3) leading to the energy balance in the structural field reads

(5) $$\frac{\partial }{\partial t}\left(\rho c_{p}T\right)=\frac{\partial }{\partial x_{i}}\left(\lambda_{s}\frac{\partial \left(T\right)}{\partial x_{i}}\right)$$

where $\lambda_{s}$ is the thermal conductivity of the solid domain.

### 4.3. Turbulence Modelling and Near-Wall Treatment

The Standard *k-ε* turbulent model was selected to close the URANS equations. It is well-known and widespread for its robustness and fair accuracy in dealing with heat exchange simulations and industrial flow. The turbulent kinetic energy *k* and the dissipation rate *ε* were obtained from the following transport equations in unsteady state:

Transport equation of k:

(6) $$\frac{\partial }{\partial t}\left(\rho k\right)+\frac{\partial }{\partial x_{i}}\left(\rho kU_{i}\right)=\frac{\partial }{\partial x_{i}}\left[\left(\mu +\frac{\mu_{t}}{\sigma_{k}}\right)\frac{\partial k}{\partial x_{i}}\right]+G_{k}+G_{b}-\rho \varepsilon -Y_{M}.$$

Transport equation of ε:

(7) $$\frac{\partial }{\partial t}\left(\rho \varepsilon \right)+\frac{\partial }{\partial x_{i}}\left(\rho \varepsilon U_{i}\right)=\frac{\partial }{\partial x_{i}}\left[\left(\mu +\frac{\mu_{t}}{\sigma_{\varepsilon }}\right)\frac{\partial \varepsilon }{\partial x_{i}}\right]+\frac{\varepsilon }{k}\left(C_{\varepsilon 1}G_{k}-C_{\varepsilon 2}\rho \varepsilon \right).$$

The generation of the turbulent kinetic energy due to the mean velocity gradients $G_{k}$ is related to the turbulent viscosity and the turbulent kinetic energy *k* and is stated as below:

(8) $$G_{k}=\mu_{t}\left(\frac{\partial U_{i}}{\partial x_{j}}+\frac{\partial U_{j}}{\partial x_{i}}\right)\frac{\partial U_{j}}{\partial x_{i}}-\frac{2}{3}\frac{\partial U_{k}}{\partial x_{k}}\left(3\mu_{t}\frac{\partial U_{k}}{\partial x_{k}}+\rho k\right),$$

where the subscript k is defined as k = 1, 2, 3.

The generation of the turbulent kinetic energy due to buoyancy for ideal gases is given as follows:

(9) $$G_{b}=-g_{i}\frac{\mu_{t}}{\rho {\mathrm{Pr}}_{t}}\frac{\partial \rho }{\partial x_{i}}$$

where $g_{i}$ is the component of the gravitational vector in the i-th direction and ${\mathrm{Pr}}_{t}$ is the turbulent Prandtl number for energy.

The eddy viscosity $\mu_{t}$ was derived from the combination of *k* and ε; it is reported as follows:

(10) $$\mu_{t}=\rho C_{\mu }\frac{k^{2}}{\varepsilon }$$

The turbulent model constants $C_{\varepsilon 1},C_{\varepsilon 2}$, $\sigma_{k},{\;\sigma }_{\varepsilon },{\;\mathrm{Pr}}_{t}$, and $C_{\mu }$ are valued as follows: 1.44, 1.92, 1, 1.3, 0.85, and 0.09, respectively [33].

In the present study, the Reynolds number in the compressible airflow domain is derived from the following expression:

(11) $$R_{e}=\frac{\rho V_{m}D}{\mu },$$

where $V_{m}$ is the fluid velocity and D is the characteristic diameter. Considering the maximum airflow velocity downstream of the valve throat, the Reynolds number is approximately 1.65 × 10^6^.

The present study adopted the scalable wall function approach (law of the wall) as the near-wall treatment, owing to the high Reynolds number in the compressible fluid. This approach is used to model the effects of the fluid boundary layer in the vicinity of the pneumatic control valve assembly, solve the URANS equations, and compute the skin friction in near-wall regions. The dimensionless length of the wall yplus (*y*^+^) is used to approximate the grid resolution near the wall. It is expressed as follows:

(12) $$y^{+}=\frac{y_{p}\rho^{1/2}\tau_{\omega }^{1/2}}{\mu },$$

where $y_{p}$ is the distance between the wall and the next adjacent sidewall cell and $\tau_{\omega }$ represents the shear stress of the wall. The particularity of the scalable wall function is to prevent degradation of the standard wall function when the grid resolution $y^{+}<11$. When $y^{+}>11$, the scalable wall function is similar to the standard, providing accurate results for grid resolution between 30 and 300 [7].

### 4.4. Discrete Methods, Computational Grid, Mesh, and Time Step Convergence Study

The numerical computations are performed based on the pressure-based solver. The finite-volume technique is chosen for the spatial and temporal discretization of the URANS equations. The pressure–velocity coupling is performed with the coupled algorithm. The convective and diffusion terms are approximated by the second-order upwind and second-order central schemes. The transient term is discretized with the fully implicit second-order scheme. All numerical calculations are processed in parallel in a high-performance cluster (Inspur TS10K, 10 nodes, Intel Xeon E5645 CPU with 24 cores and 24 GB memory per node).

The grids were generated using Ansys Fluent 19.0 (with the Fluent mesh). Polyhedral grids are adopted in the calculation fields owing to their various advantages in dealing with complex geometries: low numeric diffusion, enhancement of numerical stability, reduction of numerical diffusion, and a good approximation of gradients [16]. The local grid refinement was performed by employing curvature and proximity functions and applying four cells per gap near the valve orifice and the vent hole, where pressure and airflow gradients were more significant.

**Figure 5:**
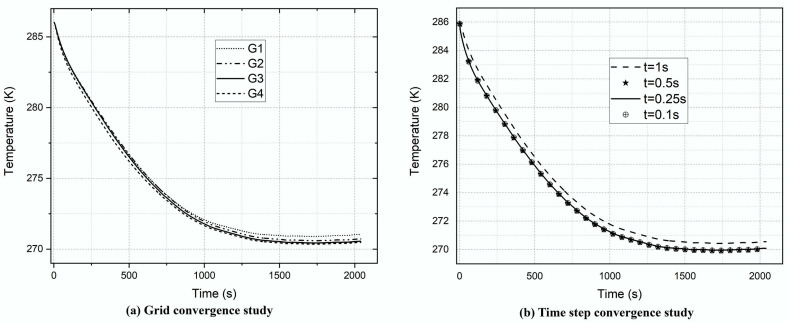
Average temperature–time evolution at the walls of the valve assembly with different grids: (**a**) for different grids; (**b**) for various time-step sizes.

**Figure 6:**
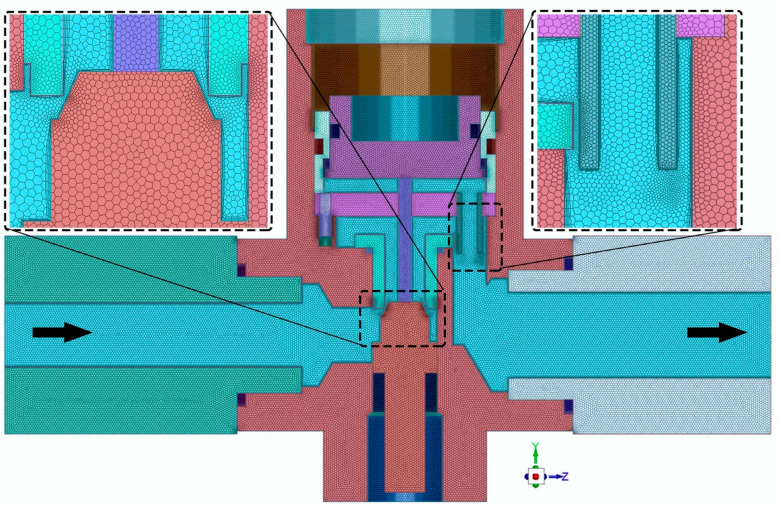
Computational grid at the YZ plane (cross-section *x* = 0).

Four grids (G) sizes—G_1_, G_2_, G_3_, and G_4_—summarized in Table 1, were generated for the mesh sensitivity analysis. Table 1 also reports the computing cost of each mesh during the calculation at the time step of 1 s. The average temperature–time distribution at the walls of the valve assembly of the mentioned grids is illustrated in Figure 5a. The grid dependency of the results is visible: the temperature–time history decreases with fine grids and is almost similar for G_2_, G_3_, and G_4_. Average temperature differences of about −16.5% and −7.42% are registered between grids G_3_ and G_2_, G_4_ and G_3_ at thermal equilibrium. The minimum grid dependency is observed between grids G_4_ and G_3_; therefore, grid G_3_ is selected to improve the computational efficiency in the subsequent simulations. The computational grid at cross-section *x* = 0 is illustrated in Figure 6.

The study of the time interval size is also conducted to find a compromise between accuracy and simulation time. Therefore, four time-step values of 1, 0.5, 0.25, and 0.1 s were selected to investigate the average wall temperature of the pneumatic valve assembly based on grid G_3_. Figure 5b shows that the flow time interval of 0.5 s decreases the average wall temperature of the pneumatic valve assemblage by 46.85% compared with the one of 1 s. Curves of the period sizes of 0.5 s, 0.25 s, and 0.1 s are approximately independent of the time length, the highest difference being −6.15% between flow times of 0.5 s and 0.25 s, and 4.36% between time steps of 0.25 s and 0.1 s. Consequently, the simulation time step of 0.25 s is retained for future simulations to save time.

### 4.5. Initial and Boundary Conditions

**Figure 7:**
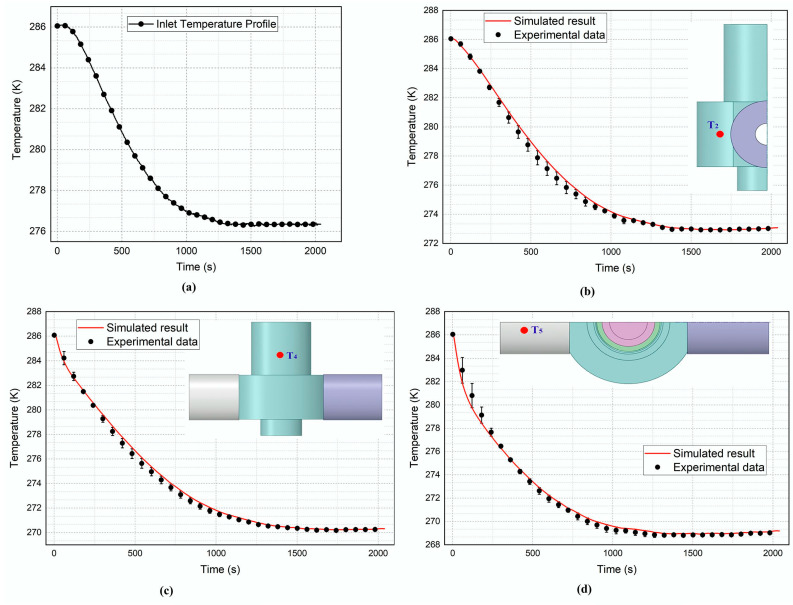
(**a**) Inlet temperature profile. Comparison between experiments and CFD results: (**b**) test point T_2_; (**c**) test point T_4_; (**d**) test point T_5_.

To complete the problem formulation, initial and boundary conditions are prescribed at each domain and at the different fields’ interfaces. The working medium was air, with a dynamic viscosity of 1.8 × 10^−5^ Kg·m·s^−1^. At the initial state, the temperature of 286.1 ± 0.1 K inside the room is measured by the thermal-resistance sensor. At the inlet boundary conditions, the pressure of 5 MPa was set. In furtherance, the compressed air inlet temperature profile shown in Figure 7a was recorded using the PT100 sensor and applied as the inlet temperature limit for the numerical simulations. The outlet boundary condition for the pressure was set at 0.3 MPa, along with the temperature of 286.1 ± 0.1 K. The initial default value of 0.001 m^2^/s^2^ was enforced for the turbulent kinetic energy k and its dissipation rate ε, respectively. The criteria for convergence of the root mean square residuals for the continuity equation—*k*; epsilon; and *x*, *y*, and *z* velocities—were set to 10^−4^ and 10^−7^ for the energy equation.

Walls boundary conditions were used at the solid and fluid domain interface. At the fluid–structure interface between the compressible flow and the inner structural field, the no-slip shear and kinematic conditions were applied at the walls [34]. Furthermore, the coupled heat transfer condition was imposed at the internal fluid–solid interface. The natural convection boundary condition was defined at the external walls of the valve assembly. The experimental room air temperature of 286.1 ± 0.1 K and a heat transfer coefficient of 0.1 W/m^2^·K were applied. The turbulent boundary conditions at the structural walls were defined as follows: k=0, ε=0, and $\mu_{t}=0$. The stainless-steel material is selected for all pneumatic control valve assemblage components at an initial temperature of 286.1 ± 0.1 K. The overall properties are resumed in Table 2.

## 5. Results and Discussions

### 5.1. Validation of the Numerical Simulations against Experiments Data

The validation of the study was carried out by experimentation, as described in session 3. The temperature history data of five different test points—namely, T_1_, T_2_, T_3_, T_4_, and T_5_—were recorded at various locations of the pneumatic valve assemblage’s outer surface. T_2_, T_4_, and T_5_ were selected and compared with the calculated temperature pattern from the numerical simulations, as shown in Figure 7b,d. The uncertainties on the computed temperatures are obtained by following the guidelines in Verification and Validation in Computational Fluid Dynamics [35].

Figure 7b displays the time variation of the measured and calculated thermal profiles at test point T_2_. Temperature discrepancies ranging from −0.1 K to 0.51 K are noticed between the measured and simulated values 24 min following the test start-up, which worsened the compliance slightly. The temperature difference falls progressively and, once the thermal equilibrium state is reached after 1500 s, its maximum value drops to 0.1 K. Despite the observed temperature fluctuations, the computational results align with the experimental data at test point T_2_.

Figure 7c shows the temperature–time trends at the T_4_ control point. The calculated thermal profile shows a temperature lapse in capturing the measured data over the 23 min of the test run. However, it is noteworthy that simulated temperatures are within 4.18% of the measurement data when the steady-state is reached after approximately 1500 s, showing fair agreement between both results.

Figure 7d compares the predicted thermal profile and data from experiments at checkpoint T_5_. It can further be observed that from the two patterns, the calculated results captured the temperature change at test point T_5_ with a maximum relative error of −6.07% at the equilibrium, indicating good conformity between the measurements and the numerical values at the checkpoint T_5_.

An overall observation of all the transient temperature–time curves indicates that they followed the same trends: decreasing over time. The temperatures at test point T_5_ were lower than those at test points T_2_ and T_4_. Moreover, the maximum temperatures at steady-state are found at test point T_2_. The agreement between measured data and simulated results endorses the methodology adopted—using the computational fluid dynamics (CFD) approach with polyhedral grids to evaluate the conjugate heat transfer (CHT) in a high-pressure pneumatic control valve assembly.

### 5.2. Analysis of Numerical Results

#### 5.2.1. Flow Performance Analysis

**Figure 8:**
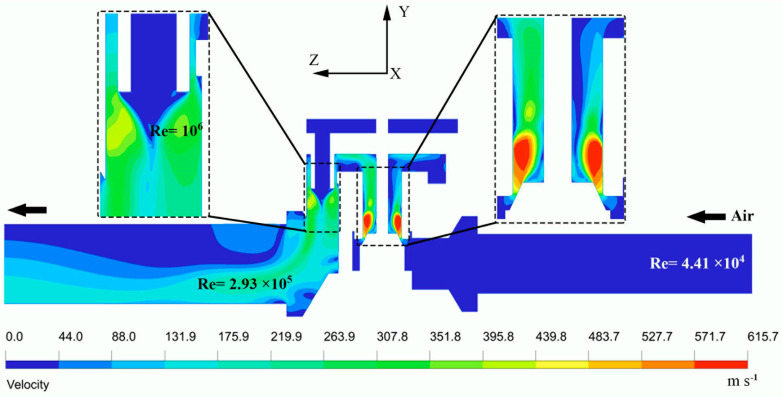
Velocity field at the cross-sections *x* = 0 of the time step t = 60 s.

Figure 8 depicts the airflow velocity field distributions at the cross-sections *x* = 0 of the 60-s time step. It can be seen that the velocity contour upstream of the valve inlet chamber is relatively constant and uniform, at approximately 13 m/s, and thus subsonic for a Reynolds number up to a value of about 1.3 × 10^4^. As the compressed air flow approaches the converging part downstream of the inlet chamber, the airflow velocity gradually increases until it reaches the speed of sound in the throat. Downstream of the throttling port, the gas expands very rapidly, the velocity speed-up from sonic to supersonic, forming a region in which the peak velocity of 615.7 m/s is reached with a high Reynolds number of 1.65 × 10^6^. At a distance of about 6 mm down the valve neck, the airflow velocity starts to decelerate until it becomes subsonic upwind of the vent hole. Further gas expansion is observed in the area between the main valve body, the vent hole, and the valve seat, where the peak velocity attains a value of approximately 439.8 m/s.

**Figure 9:**
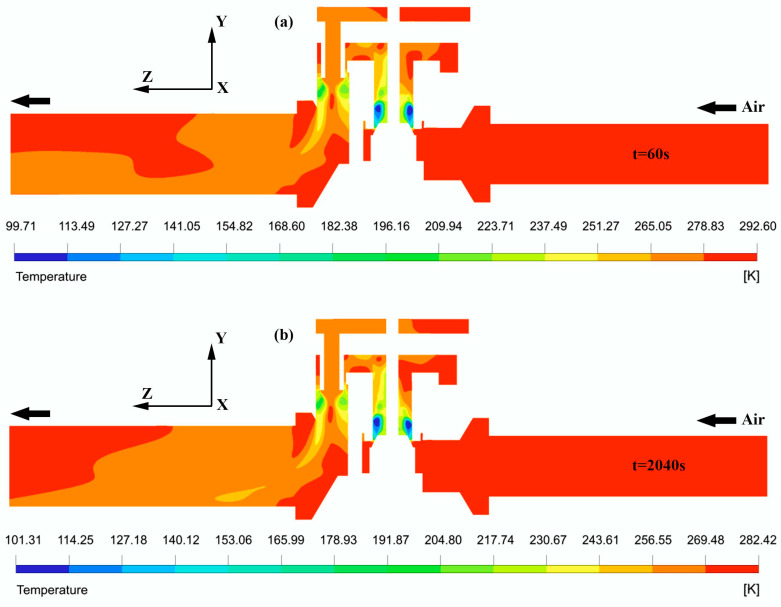
Temperature contours of airflow at various time steps: (**a**) t = 60 s; (**b**) t = 2040 s.

Figure 9a,b highlight the local temperature distributions within the compressed air domain at different time steps of 60 s and 2040 s, respectively. The temperature contour is uniform and constant upstream of the valve throat. Very low temperatures are observed downstream of the valve opening. They are a consequence of the rapid expansion of the gas through the port. At a distance of about 7 mm beyond the valve throat, the flow starts to warm up until it reaches the vicinity of the vent hole, where a second gas expansion will generate a new decrease in temperature. The overall thermal field of the flow below the valve neck is icy. Large temperature gradients are found in the flow paths of the fluid, both lengthwise and crosswise. The minimum temperature experienced at the time step t = 1 min in the airflow is 99.71 ± 0.09 K, as shown in Figure 9a. After a flow time of about 34 min, the fluid cools, and its temperature decreases by a magnitude of approximately 282.95 ± 0.09 K. This cooling also induces a reduction in the minimum temperature from 99.71 ± 0.09 K at t = 60 s to 101.31 ± 0.09 K at t = 2040 s, as indicated in Figure 9b.

#### 5.2.2. Analysis of Walls Temperature Distributions of the Valve Assemblage

**Figure 10:**
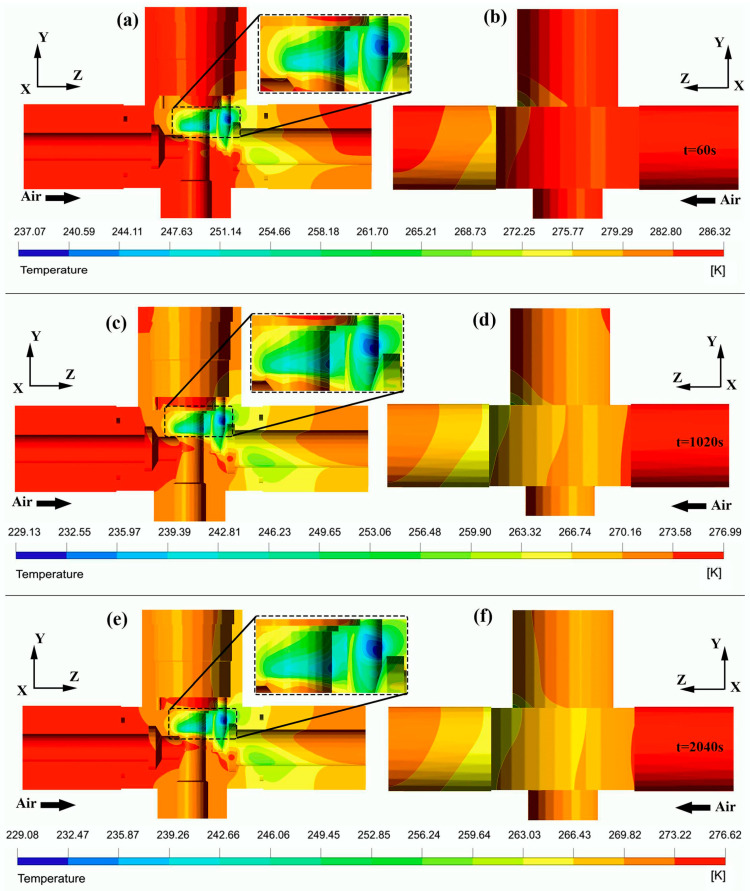
Contours of heat distribution on the walls of the valve assembly at different time steps: (**a**,**b**) t = 60 s; (**c**,**d**) t = 1020 s; (**e**,**f**) t = 2040 s.

Figure 10a,b highlight the temperature variations of the inner and outer walls of the pneumatic control valve assemblage after one minute. It can be observed that the temperatures of the inner surfaces are lower than those of the exterior surfaces due to the cooling effect of the compressed flowing gas. Both temperatures’ contours decrease in the flow direction, particularly downstream of the valve opening. Figure 10a shows the thermal shape of the internal walls of the valve body, the intake, and the exhaust connector. Significant temperature variations are observed in the *y* and *z* directions of the interior walls of the valve body, specifically between the baffle and the valve orifice, as well as those of the outlet connector after one minute, where lower temperatures are found. The minimum temperature is experienced in the vicinity of the vent hole with a value of approximately 237.07 ± 0.09 K. In Figure 10b, a rapid drop in temperature is observed between the valve exhaust chamber and the outlet connector, showing the cooling characteristic of the compressed air flowing through the structure. The minimum temperature of 272.25 ± 0.09 K occurs on the outer walls of the upper valve exit chamber and at the region below the output connector fixed to the valve body. The peak temperature recorded on the inside and outside walls is 286.32 ± 0.09 K.

Figure 10c,d show an overview of the thermal contour on the internal and external faces of the valve body, the inlet, and the outlet connector. After 17 min, the overall temperature has decreased rapidly by 282.15 ± 0.09 K. There is a significant heat difference between the two surfaces. In Figure 10c, considerable temperature variations are observed on the internal surfaces downstream of the valve neck, both in the *z*- and *y*-direction and on the outlet connector faces. In addition, it can be seen that the temperature on the inner walls of the valve underneath its opening has also undergone cooling. As shown in Figure 10d, the temperature pattern on the external surfaces of the structural domain is marked by a thermal gradient in the *z*- and *y*-direction of the valve body and the outlet connector, respectively.

Figure 10e,f highlight the inner and outer temperature field at the time step of t = 2040 s. A slight temperature decrease of about −0.1 K and 0.4 K is observed in areas of maximum and minimum temperatures, respectively, after 34 min. A gradual temperature change is witnessed on the inner and outer walls of the valve body regions located above the baffle and below its opening. In Figure 10e, the temperature on the internal surfaces of the connector outlet remains uniform and decreases slowly. Moreover, higher thermal gradients are still visible on the inner faces of the valve in the *y*–vertical and *z*–longitudinal directions. The temperature field of the exterior walls shown in Figure 10f is characterized by a considerable thermal drop in the *z*-direction. The maximum 276.62 ± 0.09 K temperature occurs on the inlet connector’s faces. In contrast, the minimum recorded temperature is observed on the interior faces of the valve body with a value of 229.08 ± 0.09 K.

#### 5.2.3. Local Entropy Generation Analysis

The local rate of entropy production per volume (${\dot{S}}_{g}{}^{\prime \prime \prime }$) in the turbulent compressed air with combined heat transfer is expressed based on the second law of thermodynamics in URANS as follows [36,37]:

(13) $$\frac{\partial \left(\rho s\right)}{\partial t}+\frac{\partial \left(\rho U_{i}s\right)}{\partial x_{j}}+\frac{\partial }{\partial x_{i}}\left(\frac{q_{i}}{T}\right)=\frac{\lambda }{T^{2}}\frac{\partial T}{\partial x_{j}}\frac{\partial T}{\partial x_{j}}+\frac{\phi }{T}>0,$$

where s represents the entropy density and $q_{i}$ the heat flux; λ is the thermal conductivity known as $\lambda_{\mathrm{cg}}$ in the ideal gas and $\lambda_{s}$ in the solid domain. The first term on the right side of Equation (13) means the entropy generation due to heat transfer (${\dot{S}}_{g,ht}{}^{\prime \prime \prime })$ in the system and the second term means the volumetric entropy generation due to the viscous dissipation (${\dot{S}}_{g,vd}{}^{\prime \prime \prime })$, defined accordingly by

(14) $${\dot{S}}_{g,ht}{}^{\prime \prime \prime }=\frac{\lambda }{T^{2}}\frac{\partial T}{\partial x_{j}}\frac{\partial T}{\partial x_{j}}$$

and

(15) $${\dot{S}}_{g,vd}{}^{\prime \prime \prime }=\frac{\phi }{T}$$

where ϕ is the viscous dissipation function given in three-dimensional cartesian coordinates by [36,38]

(16) $$\phi =\mu \{2\left[{\left(\frac{\partial U_{x}}{\partial x}\right)}^{2}+{\left(\frac{\partial U_{y}}{\partial y}\right)}^{2}+{\left(\frac{\partial U_{z}}{\partial z}\right)}^{2}\right]+{\left(\frac{\partial U_{x}}{\partial y}+\frac{\partial U_{y}}{\partial x}\right)}^{2}+{\left(\frac{\partial U_{x}}{\partial z}+\frac{\partial U_{z}}{\partial x}\right)}^{2}\;+{\left(\frac{\partial U_{y}}{\partial z}+\frac{\partial U_{z}}{\partial y}\right)}^{2}-\frac{2}{3}{\left(\frac{\partial U_{x}}{\partial x}+\frac{\partial U_{y}}{\partial y}+\frac{\partial U_{z}}{\partial z}\right)}^{2}\}.$$

**Figure 11:**
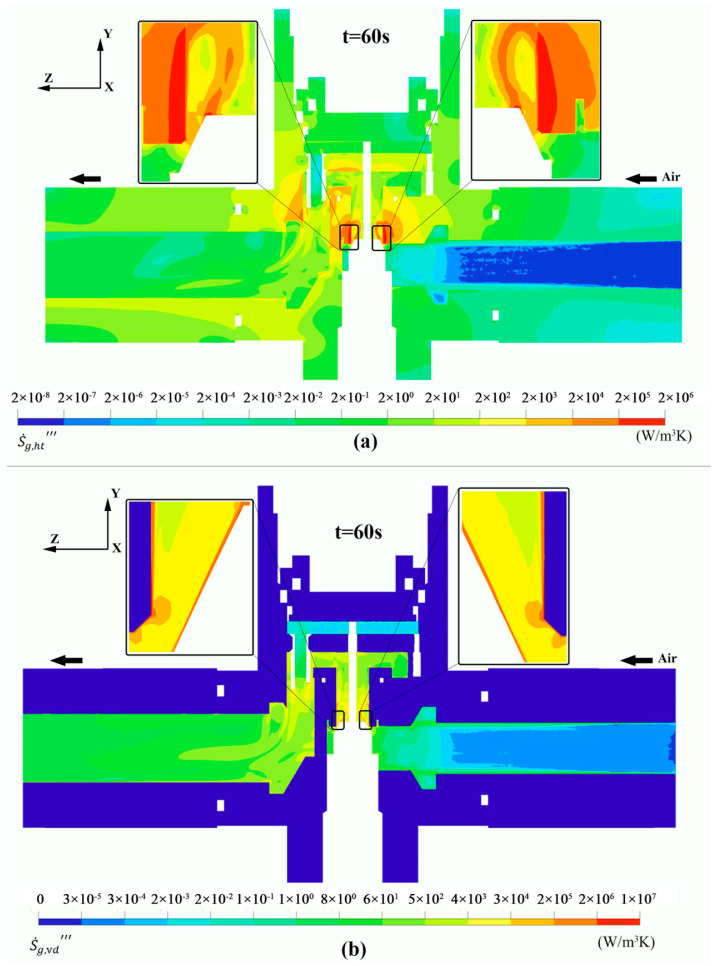
Volumetric entropy production rate by heat transfer (**a**) and viscous dissipation (**b**) at t = 60 s.

Figure 11a,b illustrate the contours of the volumetric entropy production rate by heat transfer ${\dot{S}}_{g,ht}{}^{\prime \prime \prime }$ and viscous friction ${\dot{S}}_{g,vd}{}^{\prime \prime \prime }$ in the yz plane of the control valve assembly. It can be seen from both shapes that irreversibility is mainly dominated by ${\dot{S}}_{g,vd}{}^{\prime \prime \prime }$. In Figure 11a, peak values of ${\dot{S}}_{g,ht}{}^{\prime \prime \prime }$ are observed in the valve seat domain where gas expansion occurs, indicating the presence of thermal ramps in the structural field. Moreover, ${\dot{S}}_{g,ht}{}^{\prime \prime \prime }$ is higher in airflow and the solid regions where significant temperature gradients are noticed and lower in the upstream of the flow field where small thermal slopes are detected. Optimization of the valve seat can be performed, seeking the optimal internal radius and jet flow angle to obtain an entropy-optimized design and thus reduce irreversibility losses by heat transfer in the CHT process. In Figure 11b, maximum values of ${\dot{S}}_{g,vd}{}^{\prime \prime \prime }$ are found in the thin transition layer, indicative of higher velocity differentials in the turbulent flow field adjacent to the wall. As the compressed air flows away from the valve opening, the average value of the ${\dot{S}}_{g,vd}{}^{\prime \prime \prime }$ decreases until becoming null in the solid regions where velocity slopes are zero.

## 6. Conclusions

The conjugate heat transfer (CHT) for high-pressure pneumatic valves was investigated based on the three-dimensional (3D) Unsteady Reynolds-Averaged Navier–Stokes (URANS) computational fluid dynamics (CFD) methods and a heat transfer experiment. The standard *k-ε* turbulence closure model with scalable near-wall function treatment was used to close the governing equations in the numerical simulations. Grid independence and time step convergence studies were carried out to obtain accurate solutions. A heat transfer experiment was built and data were recorded. Comparing experimental data with simulated results at checkpoints T_2_, T_4,_ and T_5_ shows good agreement. The main findings of this research are as follows:

1. CFD methods based on polyhedral grids are found to be an efficient way to investigate the CHT for a high-pressure pneumatic control valve assembly.
2. The analysis of the flow field results indicates very-high-velocity fields located in the inner airflow domain, particularly downstream of the valve neck and vent hole.
3. The compressed airflow was frigid in regions of supersonic flow, with the minimum temperature reaching 101.31 ± 0.09 Kelvin (K). Large temperatures gradients were found between the inner and outer surfaces of the valve assemblage, both in the y and z directions. The lowest temperature of about 229.08 ± 0.09 K was recorded at the inner surface of the valve body. The maximum temperature reached a magnitude of 276.62 ± 0.09 K at steady-state and at the walls of the inlet connector within 34 min.
4. The values of both thermal and viscous entropy generation rates are higher. The entropy production rate in the CHT of the high-pressure pneumatic control valve is mainly produced by viscous dissipation. An entropy-optimized system design can be obtained by seeking the valve seat’s optimum internal radius and jet flow angle to reduce irreversibility losses associated with the CHT in the high-pressure pneumatic control valve assembly.

This research lays the groundwork for several processes in and around the high-pressure pneumatic control valve assembly, including condensation and freezing. It also provides a potential baseline for thermostress analysis and optimization of the valve channel.

## Figures and Tables

**Table 1:** Characteristics of tested grids and computational costs (for a time step of 1 s).

Grids	Minimum Element Size (µm)	Maximum Element Size (mm)	Grids Number per Domain		Total Number	CPU Hour (h) at t = 1 s
			Air	Valve Assembly		
G_1_	0.18	2	370,972	932,698	1,303,670	12
G_2_		1	639,042	1,896,969	2,536,011	16
G_3_		0.55	1,256,068	7,714,024	8,970,092	23
G_4_		0.35	1,738,610	11,423,871	13,162,481	34
		Number of nodes per domain				
G_1_	0.18	2	1,086,373	3,348,487	4,434,860	
G_2_		1	1,764,883	4,752,233	6,517,116	
G_3_		0.55	2,908,212	13,002,764	15,910,976	
G_4_		0.35	3,667,464	18,033,491	21,700,955	

**Table 2:** Material properties at 286.1 K.

Material Properties	Air Ideal Gas	Stainless STEEL
Density (Kg/m^3^)	1.204	7900
Heat capacity at constant pressure (J/(Kg·K))	1004.4	460
Thermal conductivity (W/(m·K))	0.0261	16.2
Dynamics viscosity (Kg·m·s^−1^)	1.8 × 10^−5^	-

## Data Availability

Not applicable.
